# Downregulation of thromboxane A2 and angiotensin II type 1 receptors associated with aging-related decrease in internal anal sphincter tone

**DOI:** 10.1038/s41598-019-42894-4

**Published:** 2019-05-01

**Authors:** Ipsita Mohanty, Jagmohan Singh, Satish Rattan

**Affiliations:** Department of Medicine, Division of Gastroenterology & Hepatology, Sidney Kimmel Medical College of Thomas Jefferson University, 1025 Walnut St., Room #320 College, Philadelphia, Pennsylvania 19107 USA

**Keywords:** Gastroenterology, Gastrointestinal diseases

## Abstract

Aging-associated decrease in internal anal sphincter (IAS) tone (AADI) is a major contributor in the rectoanal incontinence (RI). To determine the pathogenesis of AADI, we investigated the effect of aging on GPCR activation and related downstream signaling. We particularly investigated two GPCRs that characterize IAS smooth muscle cells (SMCs): thromboxane A_2_ and angiotensin II type 1. Two groups of Fischer 344 rats (6-month-old [young group] and 26-month-old [old group]) were employed to determine the GPCR function by isometric contraction, the expressions of GPCRs, and their downstream regulatory signaling proteins (regulator of G-protein signaling 2, RGS2; GPCR Kinase 5, GRK5; and β-arrestin, Arrb2) using RT-PCR, qPCR, and western blot analyses. We used reversible biotinylation to monitor the GPCR trafficking using SMCs. Aging selectively attenuated thromboxane A_2_ and Ang II-induced IAS contraction. RT-PCR, qPCR, and WB data revealed a significant decrease in the expressions of the GPCRs and increase in the expression of RGS2, GRK5, and Arrb2. The increased GPCR internalization and decreased recycling under aging were validated by reversible biotinylation. We conclude that downregulation of GPCR, accompanied by upregulation of regulatory proteins, plays an important role in receptor desensitization and may be important underlying mechanisms of RI in certain aging patients.

## Introduction

The myogenic basal tone in the internal anal sphincter (IAS) is crucial in rectoanal incontinence (RI)^1–4^. The causes of RI are multifactorial, but a leading cause in humans is an aging-associated decrease in the IAS tone (AADI) and compromise in the fibroelastic properties (FEP) of the IAS^5–7^. Little is known about the molecular mechanisms underlying these phenomena, and this has impeded the development of a specific and safe treatment for debilitating AADI-related RI^5,8^.

Recent studies from different laboratories have shown that in animals and humans, unique myogenic properties of the IAS smooth muscle cells (SMCs) are characterized by the renin-angiotensin system (RAS) and arachidonic acid (AA) pathways, mediated through angiotensin II (Ang II) and thromboxane A_2_ (TXA_2_) by AT_1_-R and TXA_2_-R, respectively, via the G-protein coupled receptors (GPCR)^9–14^. Further studies have shown that this local control provides an extracellular trigger for the activation of RhoA-associated kinase (RhoA/ROCK), a major molecular determinant of the basal IAS tone^14–19^.

A number of studies have hypothesized that prolonged aging-related GPCR stimulation may desensitize and thus significantly decrease GPCR mediated contractility^20^. It has been speculated that such desensitization may occur either when accelerated Gα hydrolysis inhibits GPCR signaling (by conversion of GTP to GDP) or because of actual GPCR downregulation primarily via GPCR internalization, subsequently all leading to the compromised GPCR recycling^21^.

Regulators of G-protein signaling (RGS; e.g., RGS2) play an important role in inhibiting GPCR signaling by accelerating Gα hydrolysis. In addition, other regulatory proteins GPCR kinases (GRKs) that phosphorylate the C-terminal tail of the GPCR have been shown to play an important role in GPCR inactivation/internalization/downregulation in different systems, specifically by recruiting β-arrestin (Arrb), which binds to the GPCR on the internal surface^22–25^.

Despite this significant progress in the field, a logical question related to the role of GPCR regulation in relation to AADI has not been addressed: What is the role of TXA_2_-R and AT_1_-R trafficking in causing AADI? To examine this question, we carried out GPCR functional analysis by examining the effects of GPCR activation before and after their respective and selective antagonists. In addition, the studies involved expression analyses of the GPCRs (TXA_2_-R and AT_1_-R) and downward regulatory proteins (RGS2, GRK5, and Arrb2) at transcriptional and translational levels via RT-PCR, qPCR, and western blot studies. To examine the issue of receptor trafficking for the role of GPCR internalization, we focused our studies on TXA_2_-R, using reversible biotinylation approach. Present studies advance understanding of the pathophysiology of the AADI-associated RI and provide guidance for development of targeted therapy.

## Materials and Methods

### Animals and internal anal sphincter (IAS) tissues preparation

We used two distinct age groups of Fischer 344 rats: 6-month-old (young group) and 26-month-old (old group). The Institutional Animal Care and Use Committee (IACUC) of Thomas Jefferson University approved the experimental protocols. All studies were performed in accordance with the Guide for the Care and Use of Laboratory Animals of the National Institutes of Health, USA.

Rats were euthanized by decapitation, and their anal canals were quickly removed and transferred to oxygenated (95% O_2_ + 5% CO_2_) Krebs physiological solution (KPS) of the following composition: NaCl 118.07 mM, KCl 4.69 mM, CaCl_2_ 2.52 mM, MgSO_4_ 1.16 mM, NaH_2_PO_2_ 1.01 mM, NaHCO_3_ 25 mM, and glucose 11.10 mM (37 °C). The IAS SM strips (~1 × 10 mm) from the circular smooth muscle (CSM) layer were isolated as per protocol used in our previous studies^26^.

### Isometric tension measurement

Above prepared IAS SM strips from different age groups were transferred to 2 ml organ baths containing oxygenated KPS at 37 °C and employed for the isometric contraction studies as described previously^15^. All force data were monitored using force transducers (model Fort 10 g, WPI, Sarasota, FL) and Chart 4.1.2 via a PowerLab/8SP data-acquisition system (ADInstruments, Colorado Springs, CO). The IAS SM strips were allowed to equilibrate in the organ baths for 90 min for the steady development of the tone. (Spontaneous development of the tone, and its relaxant responses to appropriate electrical field stimulation parameters formulated the basis for distinguishing the IAS from the adjoining rectal SM strips^11^). The basal tone in the IAS was calculated with reference to the baseline determined at the beginning and at the end of each experiment with KPS containing 0 Ca^2+^. Cumulative concentration-response curves (CRC) for different agonists in the IAS SM strips were obtained before and after the respective antagonists and inhibitors^27–29^. We determined the CRC for U46619 (1 nM to 10 µM), and Ang II (0.1 nM to 1 µM) in the IAS in the absence and the presence of SQ29548 (0.1 µM) and losartan (0.1 µM), respectively. The range of concentrations for the agonists and antagonists was based on our previous studies^11,12,14^. The increases and decreases in IAS basal tone were expressed as absolute as well as the percentage of maximal contraction and relaxation by 100 µM bethanechol and 0 Ca^2+^, respectively.

To compare the relaxation and redevelopment kinetics for basal IAS tone of young group versus the old group, we determined the relaxing effects of Ca^2+^-free (0 Ca^2+^)-containing KPS and redevelopment of the tone with normal KPS^26,30,31^, as Ca^2+^ is the major determinant of the smooth muscle tone. Conversely, to compare the kinetics and rates of increase (g/min) in the IAS tone (or contraction), we used submaximally effective concentrations of U46619 (1 µM), Ang II (0.1 µM), and KCl (60 mM).

### Isolation of smooth muscle cells (SMCs)

SMCs from the IAS were isolated as described previously with some modifications^26,32^. Briefly, IAS tissues from the CSM layer of the anal canal were cut into 1-mm cubes and incubated in oxygenated (95% O_2_ + 5% CO_2_) KPS that contains 0.1% collagenase type I and 0.01% soybean trypsin inhibitor at 37 °C for 3 h. The cell suspension was filtered through a 500 µm Nitex mesh. The tissue trapped was carefully removed and the filtrate containing the cells was centrifuged (350 g) for 10 min at room temperature. The cells in the pellet were resuspended in DMEM with fetal bovine serum (10%) and penicillin/streptomycin (5%) in 100-mm tissue culture dishes (Corning) at 37 °C and 5% CO_2_ in an incubator with regulated humidity.

### RT-PCR and qPCR analyses

mRNAs were isolated from the IAS SMCs using Trizol reagent (ThermoFisher Scientific, Rockford, IL) according to the manufacturer’s instructions and dissolved in 40 µl of RNAase free water. The purity and concentration of total RNA were measured by a spectrophotometer at 260 nm and 280 nm. Ratios of absorption (260 nm: 280 nm) of all samples ranged from 1.8 to 2.0. First-strand cDNA synthesis was performed from 1 µg of total RNA using Sensiscript RT kit (Qiagen, Valencia, CA). Gene specific primers were designed for *TXA*_2_*-R*, *AT*_1_*-R*, *RGS*2, *GRK5*, *Arrb2*, AT_1_-R‐associated protein (*ATRAP*), hypoxanthine phosphoribosyl transferase 1 (*HPRT)*, and *GAPDH* (Table 1). All primers were procured from Integrated DNA Technologies (Coralville, IA), and 2 µl of each cDNA sample was used as a template for performing qPCR using GoTaq Green Master Mix (Promega Corp., Madison, WI) and Eppendorf Mastercycler Personal (Fisher, Allentown, PA). The PCR cycle consisted of the following sequence: 94 °C for 5 min, 94 °C for 30 s (denaturation phase), 50 °C to 60 °C for 30 s (annealing phase), and 72 °C for 1 min (elongation phase), followed by final extension at 72 °C for 5 min with a repetition of 30 cycles. Both *HPRT* and *GAPDH* were used as internal controls^33^. We observed no significant change in expression of *GAPDH* with respect to *HPRT*, hence we used *GAPDH* as our control gene for further experiments. The PCR amplicons were separated on 1.5% (wt/vol) agarose gel containing Sybersafe stain and were visualized with blue light Transilluminator Ultraslim (Transilluminators, Atkinson, NH). The relative densities of *TXA*_*2*_*-R*, *AT*_1_*-R*, *RGS2*, *GRK5*, *Arrb*,*2* and *ATRAP* were calculated by normalizing the band densities for each gene with that of *GAPDH* in RT-PCR. For qPCR assay, the relative fold changes in aging genes were normalized to corresponding young values, using 2^−ΔΔ^Ct method. All values were expressed as mean ± standard error of mean (SEM).

**Table 1:** Primers used in the PCR for amplification of cDNA encoding *TXA*_*2*_-*R*, *AT*_*1*_-*R*, *RGS2*, *GRK5*, *Arrb2*, *ATRAP*, *HPRT*, and *GAPDH* in young versus old IAS SMCs.

Primer	Sequence (5′-3′)	Accession No.	Anneal. Temp (°C)
TXA_2_-R-F	GAAGCAGACGGTTTGAGGGA	NC_005106.4	50
TXA_2_-R-R	TCAGTTTCCCCCGTGAATCG		
AT_1_-R-F	CTACAGCATCATCTTTGTGGTGGGA	NM_030985	52
AT_1_-R-R	CGTAGACAGGCTTGAGTGGGACTT		
RGS2-F	TGCGTACCCATGGACAAGAG	NM_053453.2	60
RGS2-R	CTTCCTCAGGAGAAGGCTTGAT		
GRK5-F	GCAACATGCTGCTCACCAAA	NM_030829.1	50
GRK5-R	CGAAGGGAGGGTCCAACATC		
Arrb2-F	CCACAAAAGGAACTCCGTGC	NM_012911.1 R	52
Arrb2-R	GGACGTTGACATTGAGGGGT		
ATRAP-F	TGCTTGGGGCAACTTCACTATC	NM_001007654.1	50
ATRAP-R	ACGGTGCATGTGGTAGACGAG		
HPRT-F	GCGAAAGTGGAAAAGCCAAGT	NM_012583	52
HPRT-R	GCCACATCAACAGGACTCTTGTAG		
GAPDH-F	GGCTCATGACCACAGTCCAT	NM_017008.4	60
GAPDH-R	CCCCTCCTGTTGTTATGGGG		

F: Forward segment; R: Reverse segment.

### Western blot analysis

In order to determine the total levels of TXA_2_-R, AT_1_-R, RGS2, GRK5, Arrb2, and ATRAP in the basal state in the cytoplasm, and TXA_2_-R and AT_1_-R in the particulate fractions, we used the protein lysates from IAS SMCs. The culture plates (at 80% confluency of the SMCs) were lysed in ice cold RIPA buffer (1% Nonidet P-40, 1% SDS, 0.5% sodium deoxycholate, 50 mM Tris (pH 7.4), 1 mM sodium orthovanadate, 150 mM NaCl, and 1 mM EDTA, with freshly added protease and phosphatase inhibitors cocktail), followed by centrifugation (16,000 g for 10 min) for protein estimation of RGS2, GRK5, Arrb2, and ATRAP. The protein content in the lysates were determined by using a BCA kit from Pierce (Rockford, IL). The samples were then mixed with 2x sample buffer (125 mM Tris, pH 6.8, 4% SDS, 10% glycerol, 0.006% bromophenol blue, and 2% β-mercaptoethanol) and placed on a heat block for 5 min. The proteins extracts (30 μl containing 30 μg) of each sample were separated by 10% SDS-polyacrylamide gel (for RGS2, GRK5, Arrb2, and ATRAP) using GAPDH as the loading control. These separated proteins were transferred to a polyvinylidene fluoride (PVDF) membrane by overnight incubation at 4 °C. Same samples were loaded in different gels against different proteins for GAPDH, RGS2, GRK5, Arrb2, and ATRAP.

To determine the relative distribution of TXA_2_-R and AT_1_-R in membrane versus the cytosol, the 80% confluent IAS culture plates were lysed in ice-cold homogenization buffer (10 mM Tris, pH 7.5, 5 mM MgCl_2_, 2 mM EDTA, 250 mM sucrose, 1 mM dithiothreitol, freshly added protease and phosphatase inhibitors cocktail) as described previously^6,10^. This was followed by centrifugation (100,000 *g* for 30 min, 4 °C). The supernatants were used as the cytosolic fraction, and pellets resuspended in RIPA buffer centrifuged (800 *g* for 10 min) were collected as the particulate fractions^34^. The proteins were run on the polyacrylamide gel and transferred on PVDF membrane as explained above.

The membranes were blocked in Odyssey blocking buffer (LI-COR Biotechnology, Lincoln, NE) for 1 h at room temperature followed by three washings with TBST (0.1% Tween 20). The membranes were then incubated overnight in respective primary antibodies [anti-rabbit- AT_1_-R and TXA_2_-R (1: 500); RGS2 (1: 400); GRK5 (1: 4,000); Arrb2 (1: 200); ATRAP (1: 200), and anti-mouse GAPDH (1: 5,000)] at 4 °C. The membranes were washed with TBST thrice for 10 min each and incubated with IRdye-conjugated secondary antibodies (1: 5,000) for 1 h. The membranes were then scanned using Odyssey infrared scanner. The band intensities of different proteins were analysed as ratios of GAPDH using Image J1.41 (NIH, Bethesda, MD).

### Internalization assay

The internalization of TXA_2_-R was analysed using cell-surface biotinylation approach as described by Ehlers^35^. In brief, rat IAS SMCs were biotinylated with the reversible membrane-impermeable derivative of biotin 0.5 mg/ml sulfo-NHSS-S-biotin (Pierce) for 30 min at 4 °C, as described by De Godoy and Rattan^36^. Biotin solution was replaced by fresh biotin at 15 min interval followed by washing in PBS^2+^ (PBS; 0.8 mM CaCl_2_, 1 mM MgCl_2_, pH 7.4). The cells were then incubated at 37 °C for 0 h, 1 h, and 3 h in serum free medium with or without U46619 (1 µM) to allow internalization of biotinylated cell-surface proteins. The cells were then cooled to 4 °C for 30 min to stop internalization and the remaining biotinylated proteins on the cell surface were stripped by treating the cells with quenching buffer (PBS^2+^; 100 mM glycine), followed by washing with neutralization buffer (50 mM glutathione, 75 mM NaCl, 10 mM EDTA, 1% BSA and 0.075 N NaOH).

### Recycling assay

The recycling assay was conducted to analyse the fate of the internalized pool of TXA_2_-R generated by agonist-induced internalization. Following the internalization procedure as explained above, the cells were subjected to a second round of incubation for 1.5 h or 6 h in the presence of U46619 to allow reinsertion or degradation of the internalized biotinylated TXA_2_-R. The level of surface receptor density in each step of this protocol was then analysed to determine the level of recycling of the internalized receptors to the cell surface.

To investigate the role of lysosomes and proteasomes in the degradation of the endocytosed GPCRs, we examined the effect of their respective inhibitors chloroquine diphosphate^37^ and MG132^38^. The rationale was that the degrading role of these organelles could be determined by a reversal of the downregulated GPCR (TXA_2_-R) protein at cell surface level, by incubation of old rat IAS SMC with chloroquine or MG132 (both 10 μM) for 12 h prior to the receptor recycling assay. Chloroquine was obtained from Sigma (Sigma-Aldrich, St. Louis, MO) and MG132 from Cayman (Cayman Chemical, Ann Arbor, MI). The surface receptor density was then determined following the same biotinylation technique as explained before. Following this, the cells were immediately lysed with RIPA buffer for protein extraction. Biotinylated proteins were affinity-purified from cell lysates with streptavidin-agarose (InVitrogen, Carlsbad, CA) and loaded onto SDS-10% polyacrylamide gel. Western blot analysis was then performed as described above. Biotinylation data for TXA_2_-R in the aging were expressed on the basis of maximal density at the cell surface with respect to that in young at 0 min without stripping.

### Immunofluorescence and confocal microscopy

Freshly isolated IAS SMCs were aliquoted in Eppendorf tubes, treated with biotin 0.5 mg/ml sulfo-NHSS-S-biotin, and then with U46619 in the same manner as described above. Thereafter, the nonspecific binding was blocked using 10% donkey serum and 1% BSA for 30 min, followed by incubation with Streptavidin-conjugated FITC (1: 400) in a humid chamber at 4 °C for 2 h; they were then washed three times in PBS^2+^. The cells were then smeared on a slide, air dried, and cover-slipped with ProLong Gold mounting medium (InVitrogen). Slides were kept overnight for appropriate polymerization of the mounting medium and then sealed with clear nail polish. Subsequently, the images were viewed under confocal microscope (Nikon A1R, Nikon Instruments, NY), and photographs were taken and analyzed with Image J1.41 (NIH, Bethesda, MD).

### Statistical analysis

Values represent means ± SE from at least 4 independent experiments and plotted using Prism 5.1 (GraphPad Software, La Jolla, CA). Student’s unpaired t-test was used to compare 2 different groups. Statistical significance between multiple groups was tested using one-way ANOVA. Linear regression analysis was used to identify the significance in the difference between slopes of curves. P < 0.05 was considered to be statistically significant.

## Results

### Effect of aging on the kinetics of IAS smooth muscle basal tone in young versus old rats

#### Relaxation of basal IAS tone with KPS containing 0 Ca^2+^

**Figure 1:**
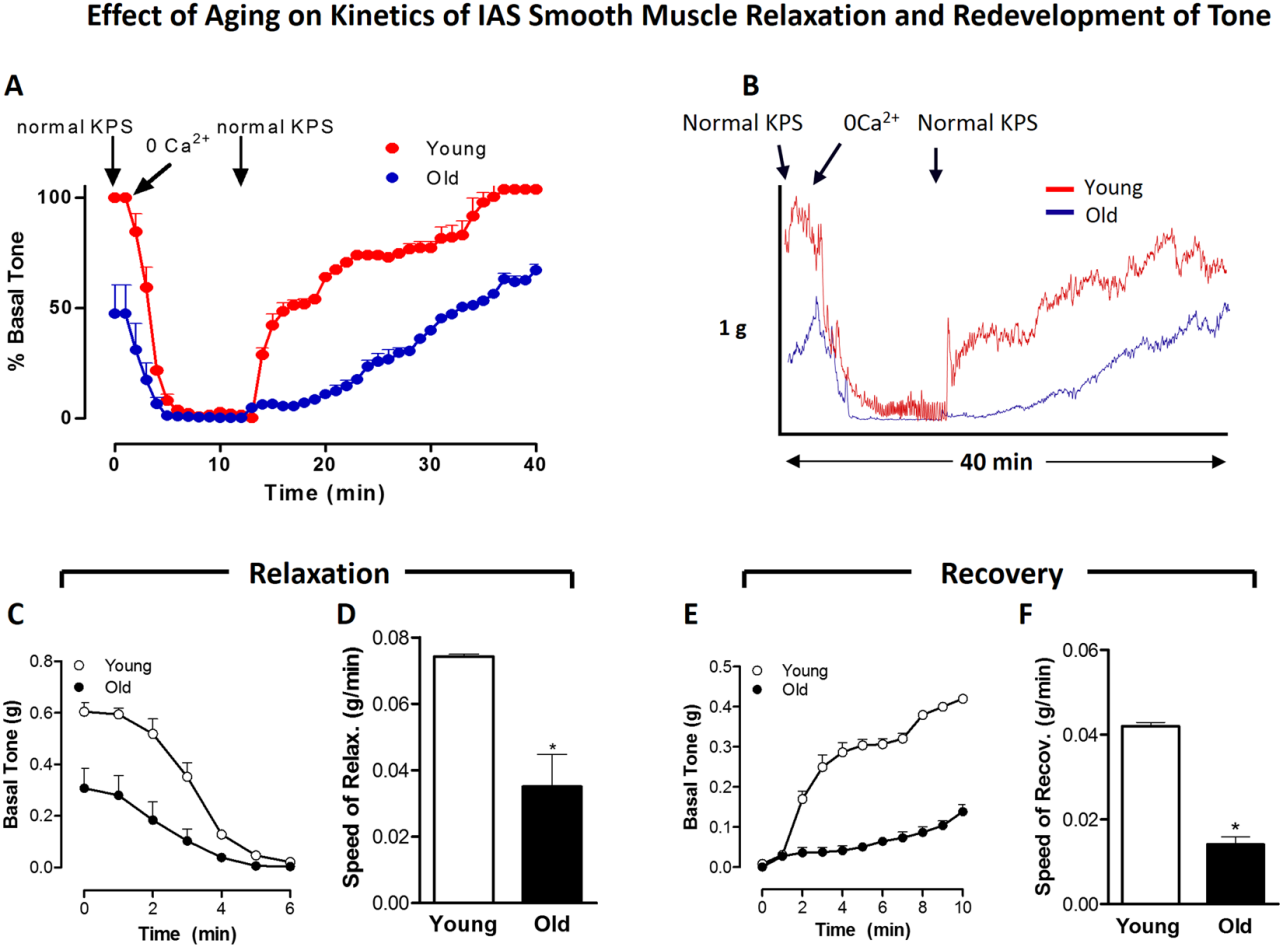
Effect of aging on kinetics of relaxation (with 0 Ca^2+^) and redevelopment of basal IAS tone, following normal KPS. Overall time-course data of IAS relaxation and redevelopment of the smooth muscle (SM) basal tone (**A**) with typical tracing (**B**), detailed analyses of decrease in absolute tone (**C**), speed of relaxation with 0 Ca^2+^ (**D**), and redevelopment of tone (**E**), and the speed of the redevelopment of the basal tone (**F**) following normal KPS. Data show that aging significantly decreases the speed of relaxation and contraction during redevelopment of tone with 0 Ca^2+^ (*P < 0.05; *n* = 4).

KPS containing 0 Ca^2+^ causes a rapid relaxation of the IAS followed by the redevelopment of its tone when replenished with normal KPS. Details of the overall time courses for these data comparing the IAS from young versus old rats are provided in Fig. 1A, and typical tracings are given in Fig. 1B. Basal IAS tone in these experiments was significantly less in the old rats (0.31 ± 0.08 g) than in the young rats (0.61 ± 0.03 g; *P* < 0.05; n = 4 in each group). Detailed analysis of the time-course for the initial relaxation reveal significant decreases in the speed of relaxation over 6 min, and for the steady redevelopment of tone over 10 min (Fig. 1C). Further comparison of relaxation rate (g/min) in the young versus old rats showed significant decrease in the speed of SM relaxation with age (*P* < 0.05; n = 4 in each group; Fig. 1C,D).

#### Redevelopment of basal IAS tone following replenishment with normal KPS

Detailed analysis of these data was carried out following the redevelopment of the basal tone from 12 min (time of sustained maximal relaxation) to 40 min (time of maximal sustained redevelopment) following normal KPS (Fig. 1A). These data (Fig. 1E,F) revealed that aging significantly attenuates the speed of redevelopment of the basal tone in the IAS (*P* < 0.05; n = 4).

### Effect of aging on the agonist (U46619, Ang II, and KCl)-induced IAS contraction kinetics in young versus old rats

**Figure 2:**
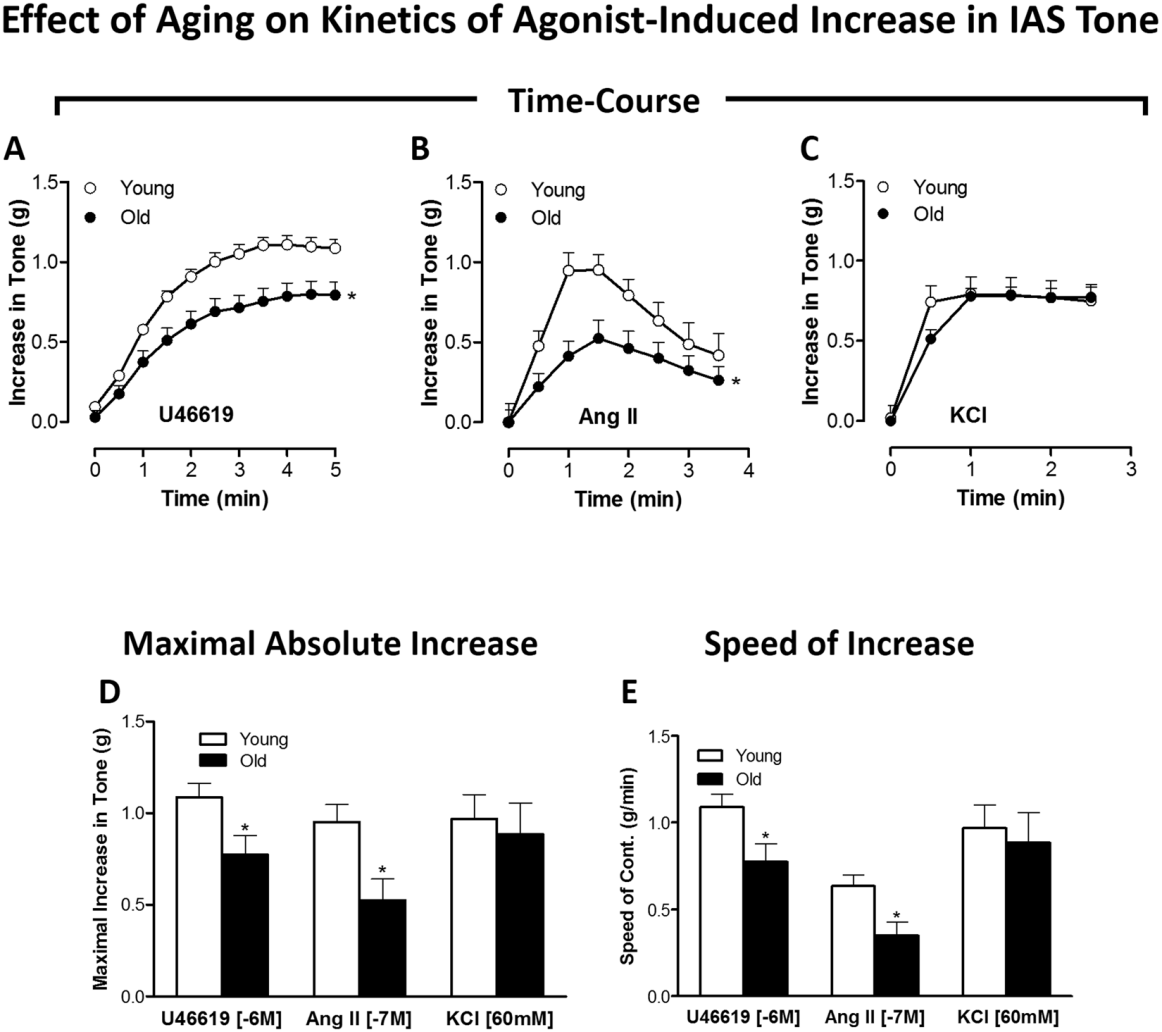
Effect of aging on kinetics of agonist-induced increase in IAS tone: (GPCR versus K^+^ -depolarization). Time-course data for the IAS contraction with U 46619 (**A**), Ang II (**B**) and KCl (**C**), and the respective maximal increases in the IAS tone (**D**). Data show that in old rats compared to young rats, the GPCR-activated increases in the IAS tone are selectively and significantly reduced (*P < 0.05; n = 8). Further data analyses (**E**) reveal that aging significantly and selectively reduces the speeds in the GPCR- but not KCl-mediated IAS contraction (*P < 0.05; n = 8).

We compared the contractile responses following GPCR activation (using U46619 for TXA_2_-R; Ang II for AT_1_-R) versus non-GPCR activation (using KCl-induced depolarization) in the IAS from young versus old rats. Herein, we examined the time-course effects of single doses of these agonists (U46619, 1 µM; Ang II, 0.1 µM; and KCl 60 mM) (Fig. 2A–C). These data, combined with in-depth calculations of maximal absolute increase (Fig. 2D) and speed of contraction (Fig. 2E), showed that aging caused significant and selective decrease in the amplitude and speed of IAS SM contraction in response to the GPCR activation and not to K^+^-depolarization (*P* < 0.05, n = 4 in each group).

Altogether, the above data suggest that aging confers significant decrease in both the IAS tone in the basal and stimulated states and in the relaxant and contractile kinetics to GPCR activation.

### Effect of aging on GPCR versus K^+^-depolarization concentration-response curves causing increase in IAS tone

**Figure 3:**
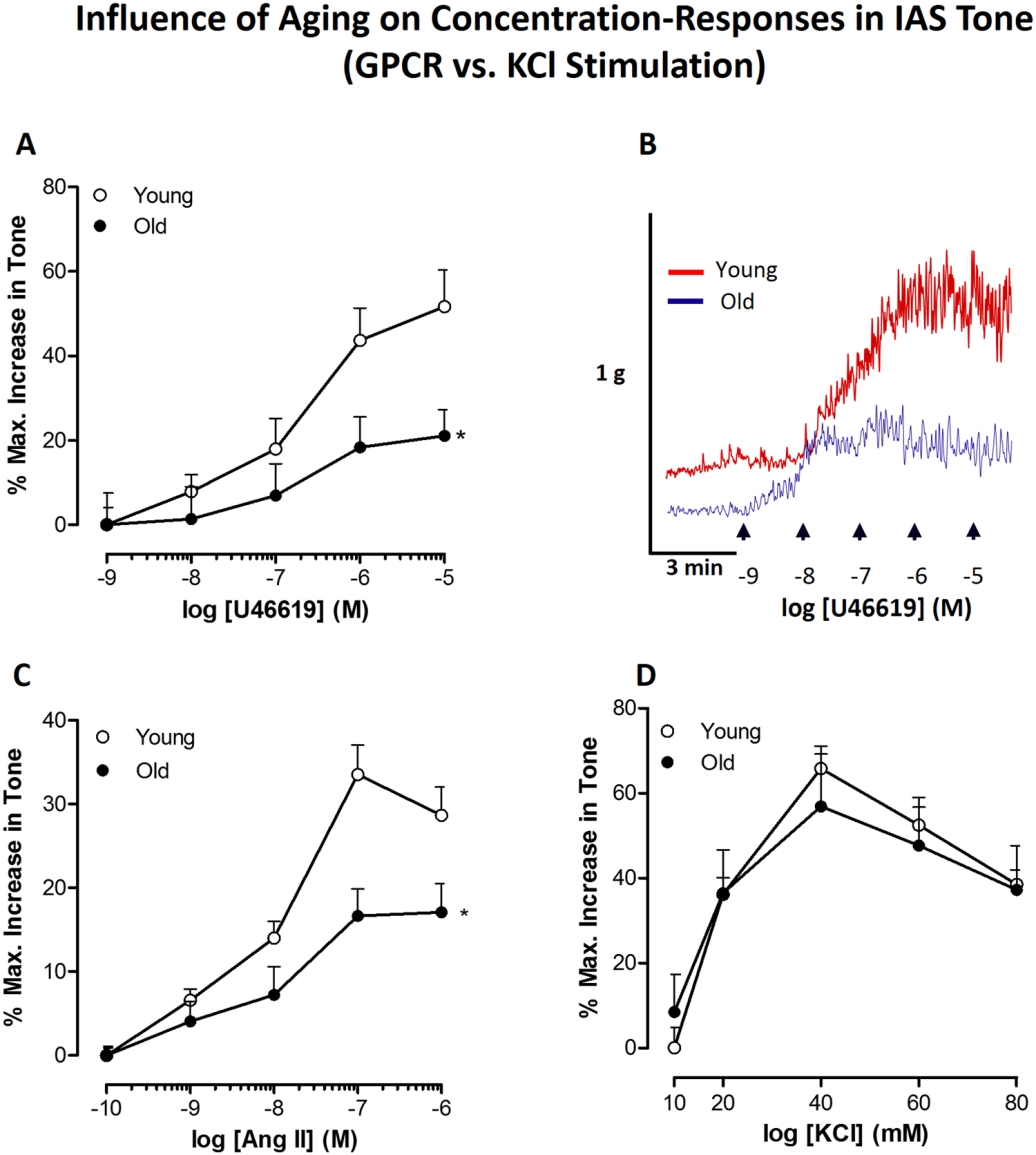
Effect of aging on GPCR (TXA_2_-R and AT_1_-R) versus K^+^-depolarization stimulated concentration-response curves (CRC). Data with concentration-response curves for U46619 (**A**), and it’s corresponding typical tracings (**B**), Ang II (**C**), and KCl (**D**) show that aging causes significant rightward shifts in the GPCR-mediated but not KCl-mediated increase in the IAS tone (*P < 0.05; n = 8).

Data in Fig. 3A–C revealed significant rightward shifts in the concentration-response curves (CRC) for U 46619 (0.1 nM to 10 µM) and Ang II (1 nM to 1 µM) in the old versus young IAS (*P* < 0.05, n = 8). These data showed a significant decrease in maximal responses and potencies in the old rat compared to the young rat IAS (RIAS). However, the data revealed no significant differences in KCl-induced CRC (10 to 80 mM) in young versus old RIAS (Fig. 3D). Representative tracings of the force experiments with U46619 are given in Fig. 3B.

### Effect of aging on the potency of selective antagonists SQ29548 and losartan in antagonizing the TXA_2_-R and AT_1_-R activation

**Figure 4:**
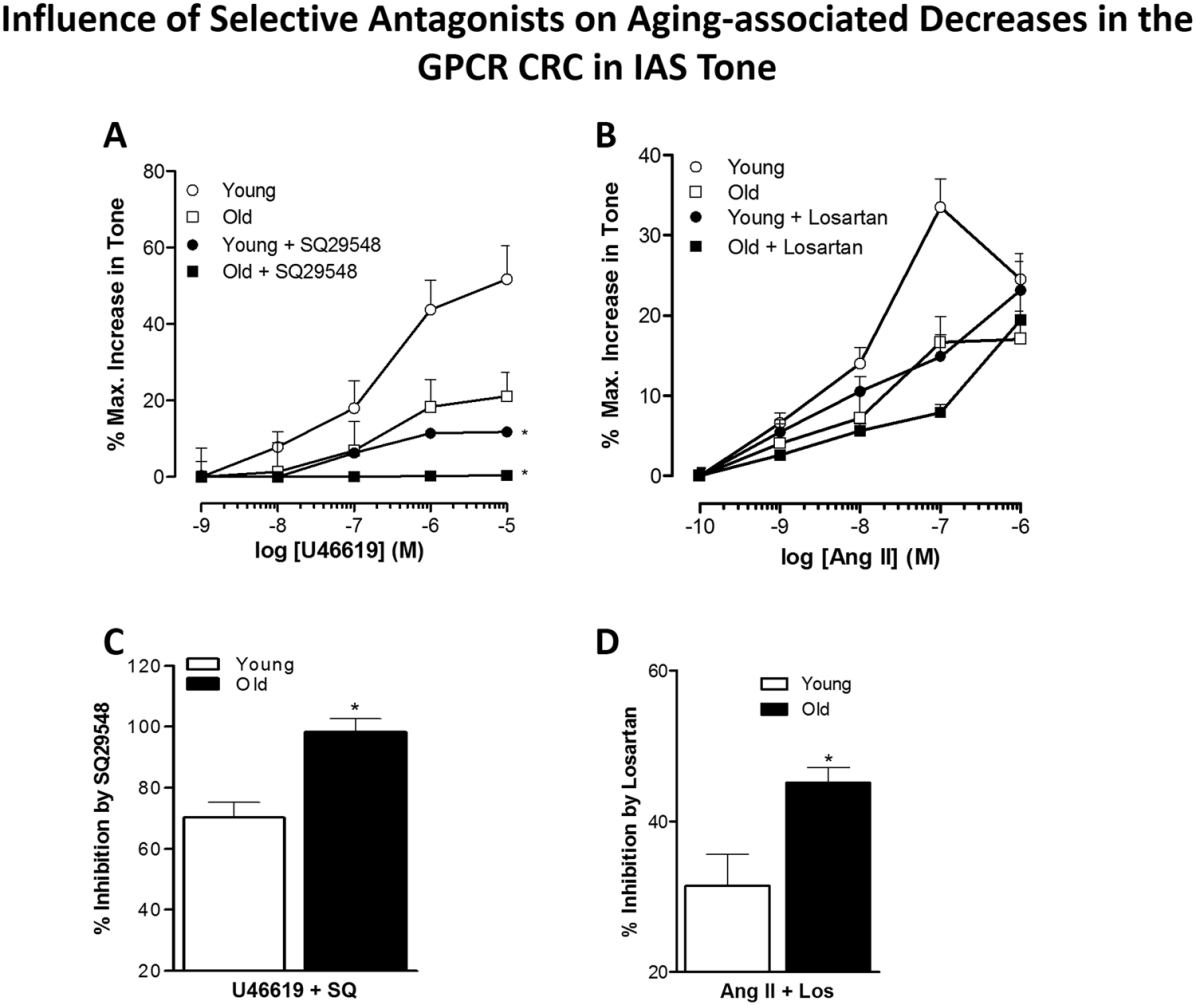
Effect of aging on the potency of TXA_2_-R and AT_1_-R antagonists. Comparison of concentration-response curves for U46619 in the presence of SQ29548 (**A**) and for Ang II in the presence of losartan (**B**) show that aging significantly increases the blocking effects of the selective antagonists of their respective GPCR agonists (**C**,**D**) (*P < 0.05; n = 8). These data imply that aging may lead to a decrease in the GPCR population.

These experimental data showed that aging significantly increased the potencies of SQ29548 and losartan in antagonizing the increase in the IAS tone caused by GPCR activation (*P* < 0.05, n = 8; Fig. 4A,B). This concept was further confirmed by comparing how these antagonists inhibited GPCR responses (Fig. 4C,D). These data indicated a trend towards decrease in GPCR population in old versus young IAS, which may be partly responsible for the observed decreased responses to GPCR activation.

### Expression analyses of GPCR in young versus old RIAS through RT-PCR, qPCR, and western blot

**Figure 5:**
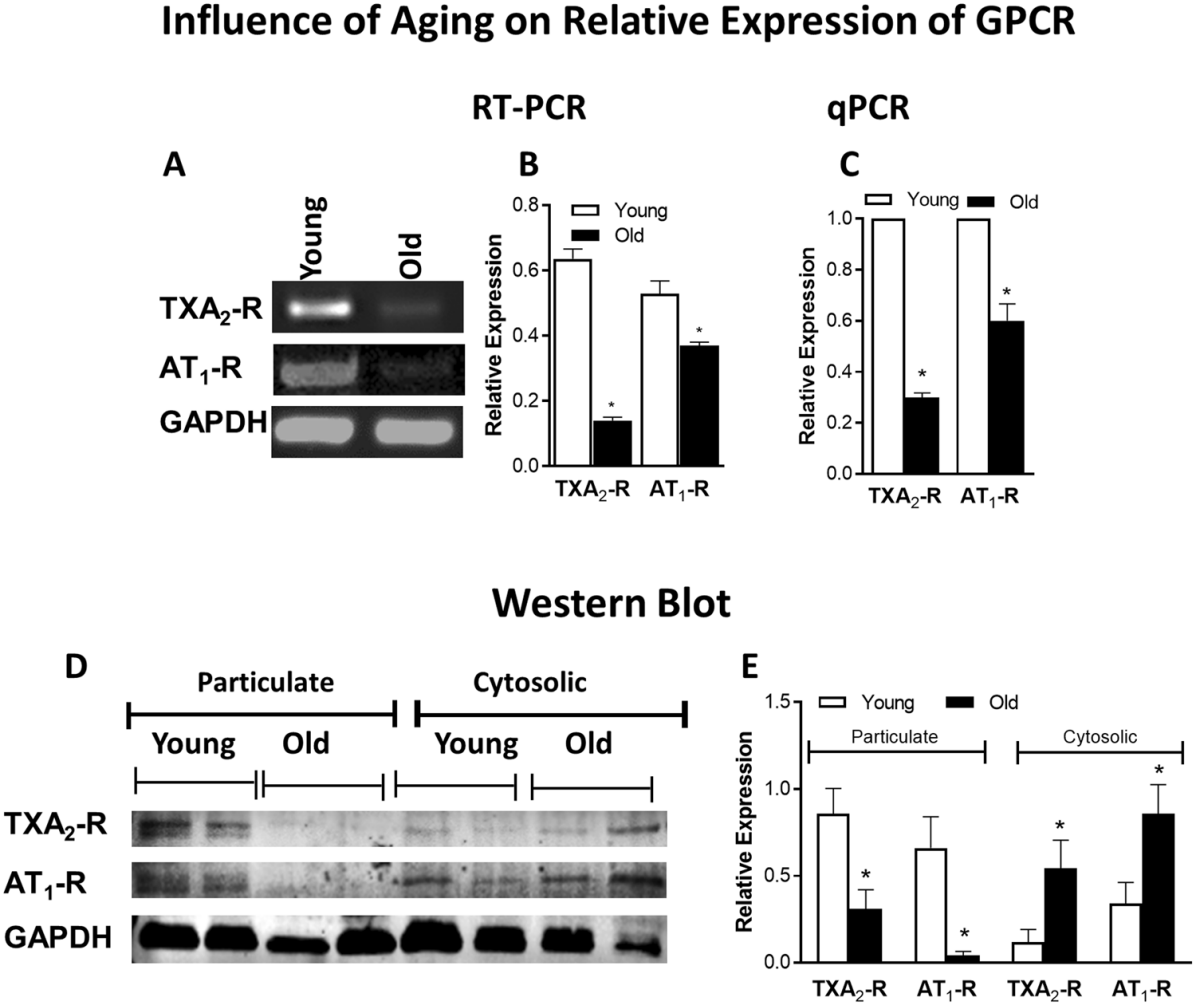
Influence of aging on relative expression of GPCR (TXA_2_-R and AT_1_-R) through RT-PCR, qPCR, and western blot (WB). A significant decrease in mRNA expression of TXA_2_-R and AT_1_-R under aging is evident through RT-PCR (**A**,**B**) and qPCR (**C**) in the RIAS from the old versus young rats (*P < 0.05; n = 4). WB analyses (**D**,**E**) also show a significant decrease in particulate fraction as well as increase in cytosolic fraction of TXA_2_-R and AT_1_-R expression under aging (*P < 0.05; n = 4). All values are compared with respect to GAPDH.

RT-PCR and qPCR analyses showed significantly low expressions of AT_1_-R and TXA_2_-R at gene level in the IAS of the old versus young RIAS (*P* < 0.05; n = 4; Fig. 5A–C).

Western blot data resembled the PCR data in showing a significant decrease in the expression of active AT_1_-R and TXA_2_-R as determined in the particulate fractions of old versus young RIAS (*P* < 0.05; n = 4; Fig. 5D,E). Full gel/blot images for AT_1_-R, TXA_2_-R, and GAPDH are provided as Supplementary Data (Figs S1 and S2).

### Expression analyses of GPCR downstream signaling proteins in young versus old RIAS through RT-PCR, qPCR, and western blot

**Figure 6:**
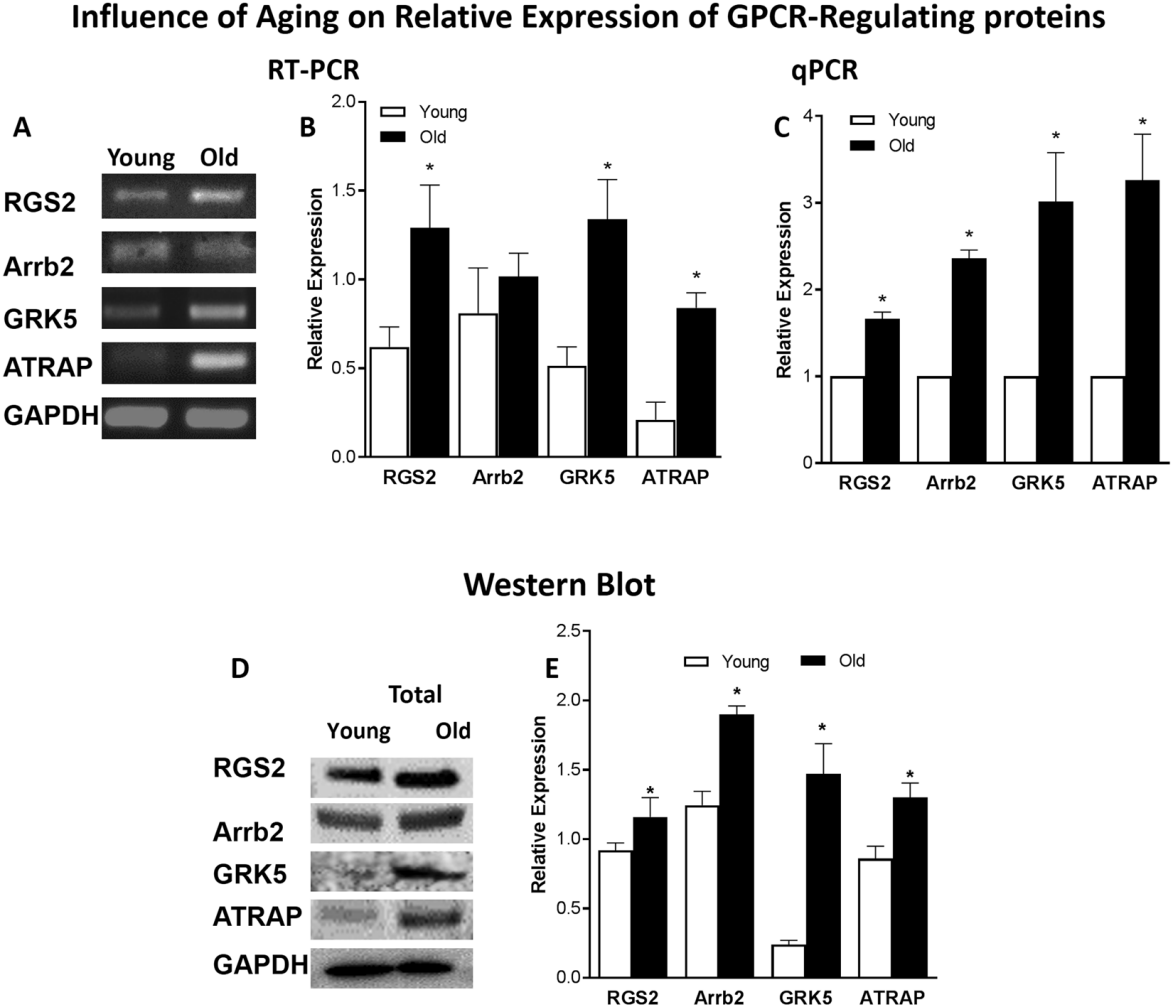
Influence of aging on relative expression of GPCR signaling regulatory proteins (RGS2, Arrb2, GRK5, ATRAP) in RIAS. RT-PCR (**A**,**B**), qPCR (**C**), and WB analyses (**D**,**E**) show significant increase in the expressions of GPCR signaling regulatory proteins, RGS2, Arrb2, GRK5, ATRAP in the aging as compared with the young IAS SMCs (*P < 0.05; n = 4). Herein, those values are compared with respect to GAPDH for RT-PCR and WB, whereas qPCR values are normalized with respect to the data in young animals.

RT-PCR and qPCR analysis showed that mRNA expression of regulatory proteins RGS2, GRK5, Arrb2, and ATRAP was significantly higher in the old versus young RIAS (*P* < 0.05; n = 4; Fig. 6A–C). Translational data examined via western blot analyses also revealed that RGS2, GRK5, Arrb2, and ATRAP protein levels in the total lysate of RIAS were significantly increased in the old versus the young group (*P* < 0.05; n = 4; Fig. 6D,E). Even though, the expression of Arrb2 was unaltered under aging in RT-PCR, quantitative PCR showed a significant increase in it’s mRNA expression level (*P* < 0.05; n = 4; Fig. 6C). Full gel/blot images for RGS2, GRK5, Arrb2, ATRAP, and GAPDH are provided as supplementary data (Figs S3 and S4).

### Effect of aging on TXA_2_-R trafficking using reversible biotinylation in young versus old

**Figure 7:**
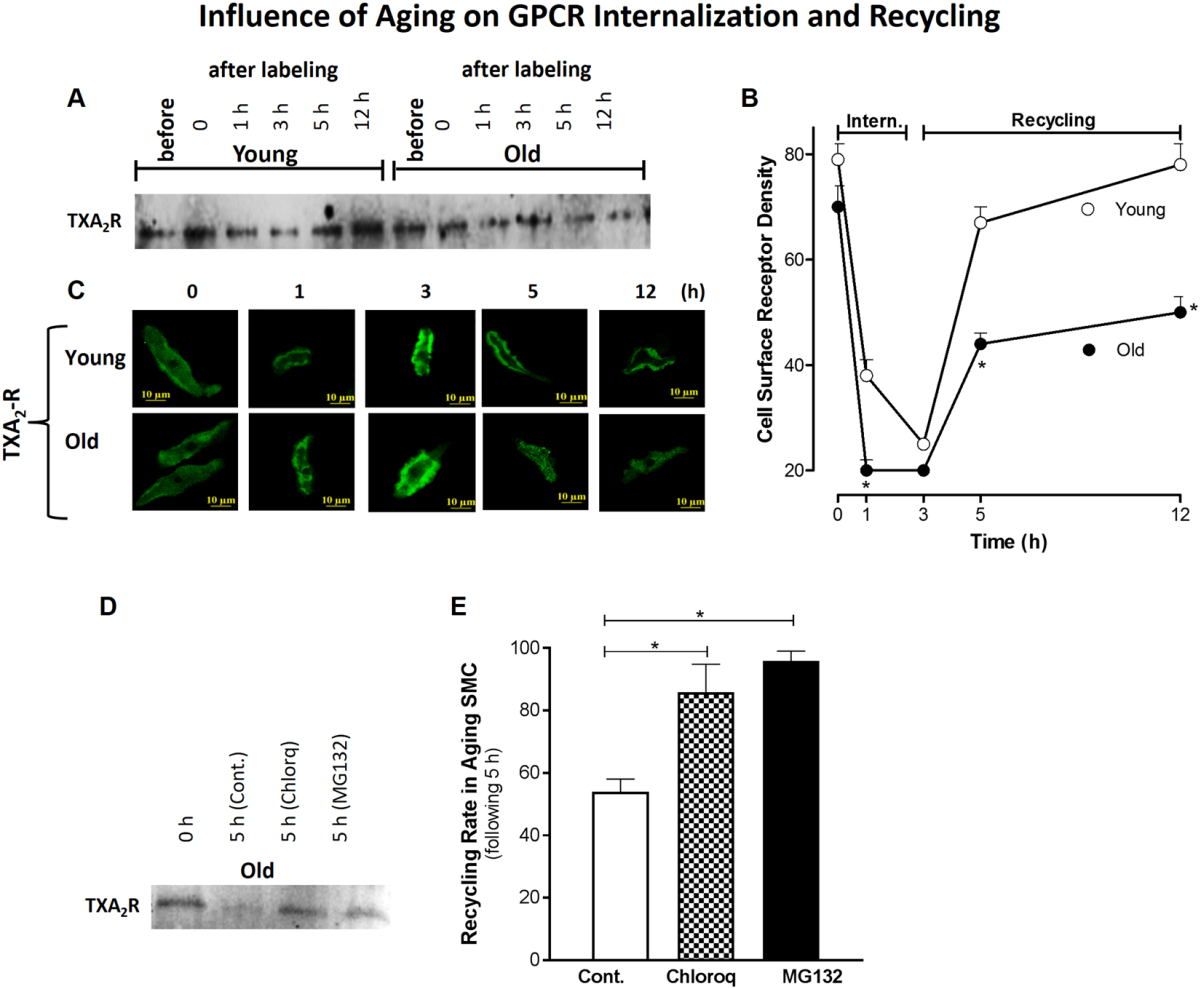
TXA_2_-R trafficking in young versus old RIAS SMC by reversible biotinylation. IAS SMC membranous receptors were labeled using biotin and incubated with their respective agonists at 37 °C for varying amounts of time, after which the cells were lysed with RIPA. Biotinylated proteins affinity-purified from cell lysate using streptavidin-agarose monitored via WB (**A**,**B**) and confocal microscopy (**C**) show that aging induces faster internalization at early phase of receptor trafficking (represented after 1 h incubation) and slow recycling of cell-surface receptors. The receptor recycling rate was significantly increased (representing rescue from internalization) following pre-incubation of the aging RIAS SMC with chloroquine (lysomotoropic agent) and MG132 (proteasomal inhibitor) (both 10 μM), as determined by normalizing the cell surface receptor density at 0 h and 5 h time points (**D**,**E**) (*P < 0.05; n = 4).

Since TXA_2_-R has been shown to play a predominant role in the basal IAS tone^14^, to examine details of the cellular trafficking, we concentrated our studies on the TXA_2_-R. The fate of TXA_2_-R (following respective GPCR activation) as a function of age was tracked down in the IAS SMC using reversible biotinylation following a time-course for serial internalization (0, 1, 3 h before) and recycling (1.5, 6 h after) (Fig. 7A,B). Data showed that TXA_2_-R density decreased significantly in old versus young RIAS SMC at 1 h (suggesting faster internalization) that plateaued at 3 h (*P* < 0.05; n = 4). Confocal microscopy data (C) also revealed that aging caused significantly less and slower recycling of the GPCR in the IAS SMC as determined at 5 h (*P* < 0.05; n = 4). Receptor biotinylation showed that MG132 and chloroquine (both 10 µM) in the aging RIAS SMCs during a course of 5 h period (3 h internalization + 0.5 h incubation at 4 °C for internalization stoppage + 1.5 h recycling) significantly increased receptor recovery inhibiting receptor loss of TXA_2_-R (*P* < 0.05; n = 4; Fig. 7D,E). Full blot images for TXA_2_-R trafficking in young versus old, and receptor recycling rate in the presence of MG132 and chloroquine through receptor biotinylation are provided in Supplementary Fig. S5A,B.

## Discussion

**Figure 8:**
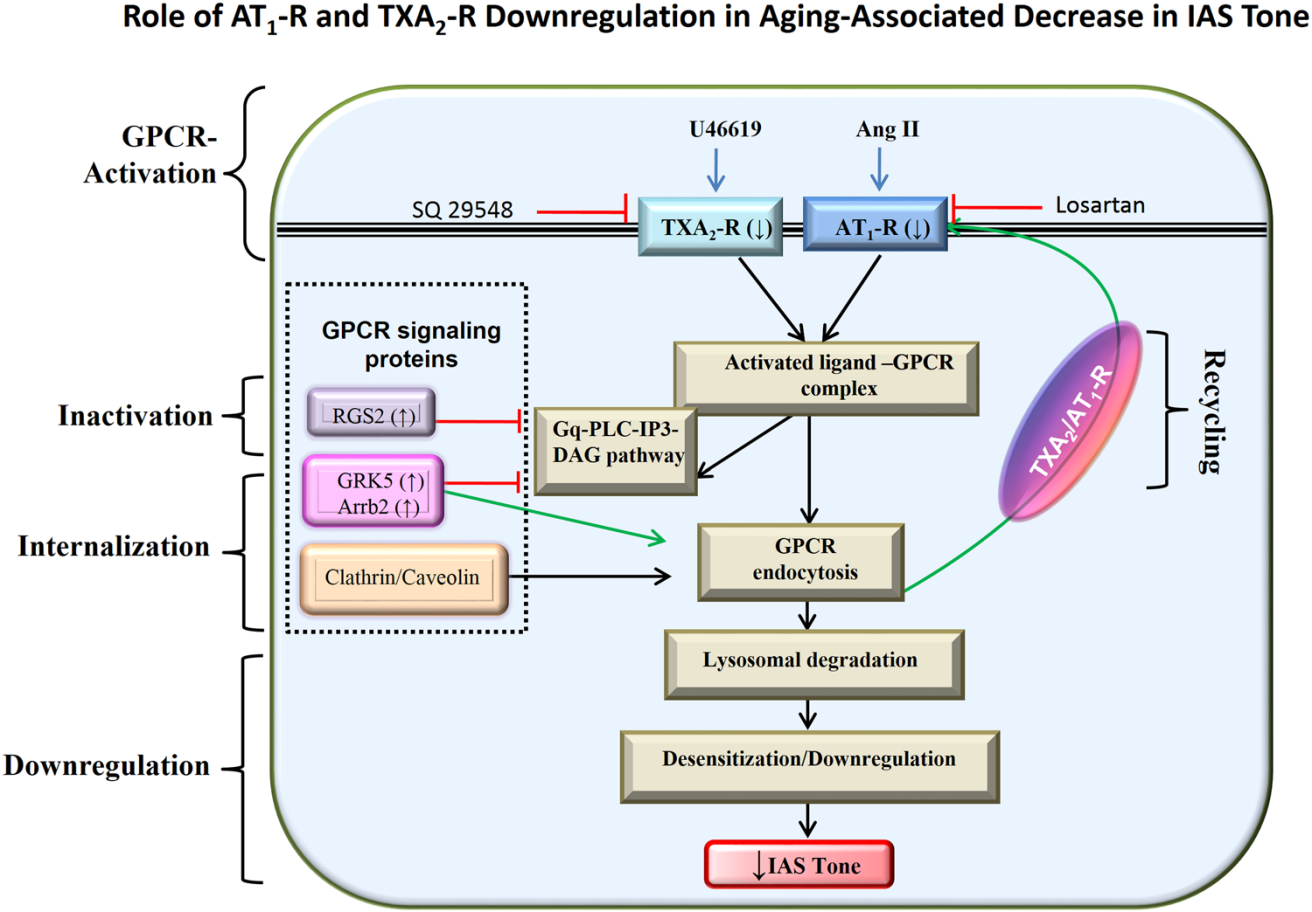
Schematic representation of TXA_2_-R and AT_1_-R downregulation in aging-associated decrease in IAS tone. Normally, GPCRs such as TXA_2_-R and AT_1_-R bind to the respective endogenous ligands responsible for the IAS tone to stimulate smooth muscle contraction through the IP3-DAG cascade, and then with the action of regulatory proteins they may be endocytosed and recycled back to the membrane. However, an increase in the Regulator of G-protein Signaling (RGS2) during aging may inactivate this GPCR signaling by acceleration of Gα hydrolysis, converting GTP to GDP. GPCR kinase5 (GRK5) by phosphorylating cytoplasmic tail of receptor stimulates receptor internalization via β-arrestin (Arrb2). These events may lead to downregulation of receptors, and combined with the GPCR lysosomal/proteasomal degradation and slower recycling may lead to functional desensitization of GPCR signal transduction by reducing the number of membrane receptors. These events may be primarily responsible for the aging-associated decrease in the IAS tone by compromise in Ca^2+^/calmodulin/MLCK/RhoA/ROCK.

These studies for the first time demonstrate the role of autocrine GPCR dysregulation in aging-associated decrease in IAS tone (AADI), as illustrated in Fig. 8. The studies reveal that aging leads to: 1) A decrease in the GPCR (TXA_2_-R and AT_1_-R)-mediated IAS tone, and in the fibroelastic properties (FEP) of the SM which correlate with decrease in the GPCR expression at gene and protein levels; 2) An increase in the expression of GPCR downstream signalling regulatory proteins, RGS2, GRK5, and Arrb2; and 3) Downregulation and compromise in the GPCR recycling leading to a decrease in the IAS tone.

Our studies build on previously published studies^6,39^ to show that AADI may be mediated by decrease in the response to GPCR (TXA_2_-R and AT_1_-R) activation. This effect was established by the rightward shifts in the IAS contractile response to U46619 (TXA_2_ stable analogue) and Ang II. The specificity of these age-related effects was established by the lack of effects of aging on K^+^-depolarization-induced increase in the IAS tone, and by the competitive antagonism of the GPCR effects by their respective and selective antagonists, SQ29548 and losartan. These data imply an increased potency of these antagonists under aging. These data are consistent with the findings in the aging urinary bladder detrusor smooth muscle reporting a selective attenuation of the GPCR effects^40–42^.

Present data further revealed an aging-associated compromise in the FEP of the SM, as shown by the rightward shifts in the time-course data and rates of SM contractility following the GPCR activation with U46619 and Ang II. Interestingly, similar data for the effects of aging on time-course of relaxation and contractile responses, and their corresponding speeds, were obtained for the relaxation and redevelopment of the basal tone following 0 Ca^2+^ and its replenishment with normal KPS, respectively. Such data are in agreement with the previous data in other SM systems during aging^43^. It is conceivable that decrease in the basal tone and compromise in the FEP of the IAS, with respect to decreased GPCR activation during aging, is caused by the downregulation of the Ca^2+^/calmodulin/MLCK/RhoA/ROCK molecular control mechanism within the SMCs^14,17,19,30,39,44^.

A plausible explanation for the GPCR-associated AADI is downregulation of GPCR at the cellular membrane and the underlying downstream signaling, as suggested previously in different SM systems^43,45^. In support of this, western blot and PCR studies demonstrate that aging causes a significant decrease in the expressions of TXA_2_-R and AT_1_-R in the particulate fraction and mRNA in the IAS SMC. Interestingly, we observed an increased translocation of both TXA_2_-R and AT_1_-R from particulate to cytosol under aging, suggesting compromised response from the agonist stimulated Ca^2+^ sensitization. These data provide credence to the earlier hypothesis that TXA_2_ and Ang II biosynthesis are responsible for the basal myogenic IAS tone in animals and humans^6,10,14,26,34^.

Whether the decrease in GPCR downregulation during aging is solely responsible for the observed AADI or whether AADI also involves the post-activation GPCR signaling remains unclear. Typically, a balance between the regulation of GPCR activation, internalization (endocytosis), degradation, and recycling events may determine the final functional outcome of GPCR efficacy. This issue is relevant in the pathophysiology of AADI, since an inhibition of autocrine GPCR responsiveness may lead to the AADI and thus RI.

There is substantial literature to suggest that the regulators of G-protein signaling (RGS), especially RGS2, GPCR kinase (GRK), and β-arrestin (Arrb), play an important role in regulating post-activation GPCR signaling^21,46,47^. Accordingly, GRKs phosphorylate agonist-activated GPCR and then Arrb bind to these phosphorylated GPCR, leading to receptor internalization and desensitization^48^. Consequently, prolonged interaction of Arrb with GPCR may cause sustained downregulation. In support of these concepts, our data show that aging causes a significant increase in the expression profile of the above-stated downstream negatively operating GPCR signaling proteins RGS2, GRK5, and Arrb2, as determined via WB and RT-PCR and qPCR. Similar data has also been reported in other systems^22–25^. Whether GPCR downregulation is related to the further GPCR degradation in lysosomes or to distinct recycling pathways is not yet known. The marked increase in mRNA and protein expression of RGS2 that we found in our studies resembles the κ opioid receptor desensitization that has been documented in diabetic rat cardiomyocytes^49^. Present data implicate the presence of a phosphorylation-independent desensitization mechanism in the aging IAS, as reported in different systems^50,51^. Parallel studies revealing the increased expression profile of ATRAP in aging suggests an enhanced AT_1_-R internalization and desensitization that reduces AT_1_-R signaling in the IAS as reported in other systems^52^. For the present studies, GRK5, Arrb2, and RGS2 were selected as the regulatory GPCR signaling proteins of investigation because of their significant role in the regulation of the GPCR signaling in the gastrointestinal smooth muscle contractility^53^.

Our findings may explain that the age-related attenuated GPCR efficacy in AADI occurs due to increased expression of RGS2, GRK5, and Arrb2 leading to a higher basal phosphorylation and desensitization of the GPCRs. This concept is supported by earlier data elsewhere^54^. Future studies using knockout models of RGS2, GRK5, and Arrb2 in IAS followed by their correlations with the basal tone and contractile responses may further delineate the pathogenesis and the extent of involvement of these regulatory proteins in GPCR desensitization, endocytosis, or degradation in the AADI.

To examine, the role of GPCR endocytosis in intracellular trafficking for the compromised GPCR activation in AADI, we performed time-course studies on TXA_2_-R using a reversible biotinylation approach combined with western blot and confocal microscopy. These data reveal a faster endocytosis and slower recycling of the GPCR in aging IAS SMCs. These data may point to a compromise in recycled receptor density in the aging SMC and indicate endosomal degradation and desensitization of the GPCR. A partial reversal of these processes in the aging IAS SMCs using proteasomal inhibitors (e.g., MG132) and lysosomal inhibitors (e.g., chloroquine) further supports this concept^37,38^.

In conclusion, AADI may be partly attributed to TXA_2_-R and AT_1_-R downregulation via desensitization, lysosomal degradation, and compromised recycling of the GPCRs. RGS2, GRK5, and Arrb2 may play an important role in this process. Our findings on the molecular regulation of the GPCR in AADI have direct implications for understanding the pathophysiology of rectoanal incontinence in the elderly associated with the IAS dysfunction and for guiding the development of therapeutic interventions.

## Supplementary information

Supplementary information

## Data Availability

All data generated and analyzed during the current study are available from the corresponding author on reasonable request.
