# Supplementary material for: Downregulation of thromboxane A2 and angiotensin II type 1 receptors associated with aging-related decrease in internal anal sphincter tone
Source: Sci Rep. 2019 May 1;9:6759. doi: 10.1038/s41598-019-42894-4 (PMC6494869; doi:10.1038/s41598-019-42894-4)
Supplement: Supplementary file 1 — Supplementary information [file 41598_2019_42894_MOESM1_ESM.pdf]

Supplementary Information

Downregulation of thromboxane A2 and angiotensin II type 1 receptors associated with aging-related decrease in internal anal sphincter tone

Ipsita Mohanty, Jagmohan Singh and Satish Rattan

Department of Medicine, Division of Gastroenterology & Hepatology, Sidney Kimmel Medical College of Thomas Jefferson University, Philadelphia, Pennsylvania 19107

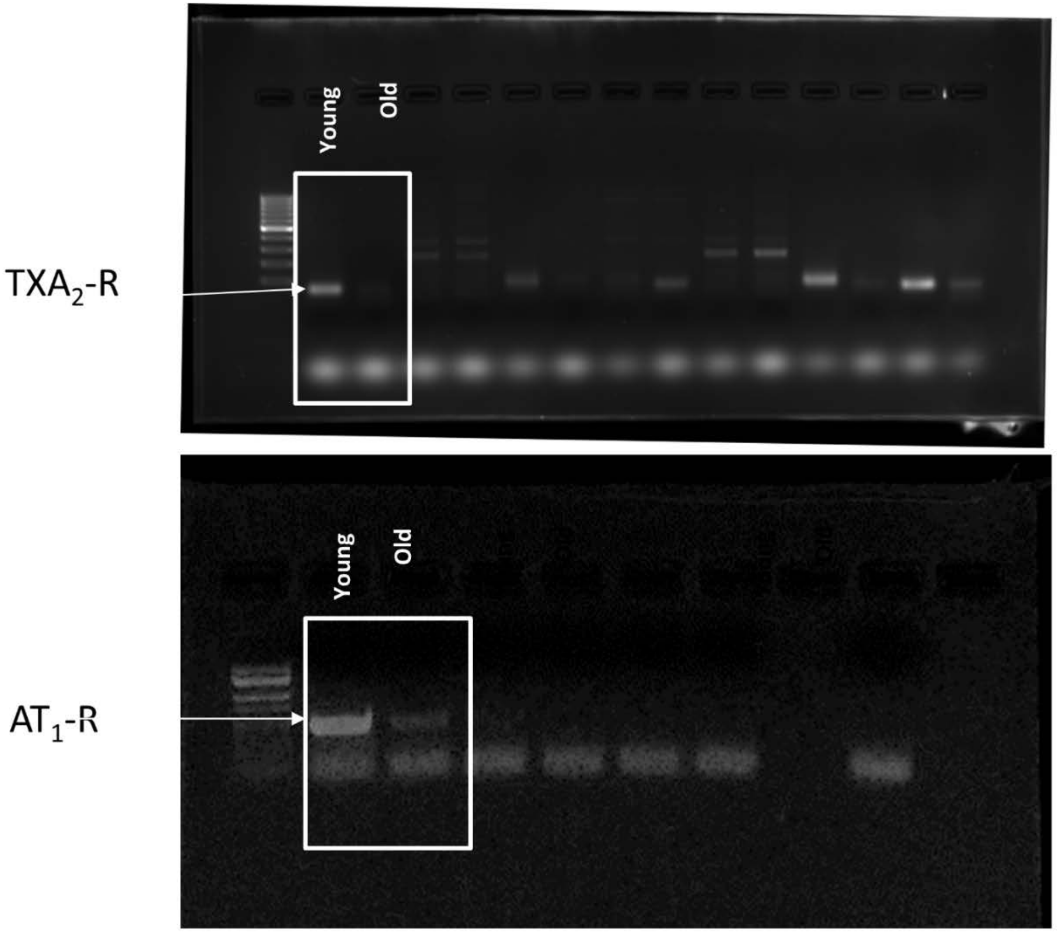

Figure S1. Full gel images of TXA_2-R (top panel) and AT_1-R (bottom panel) in young vs. old through RT-PCR. RT-PCR analyses show significant decrease in TXA_2-R and AT_1-R mRNA expression under aging. Values are compared with respect to GAPDH**.**

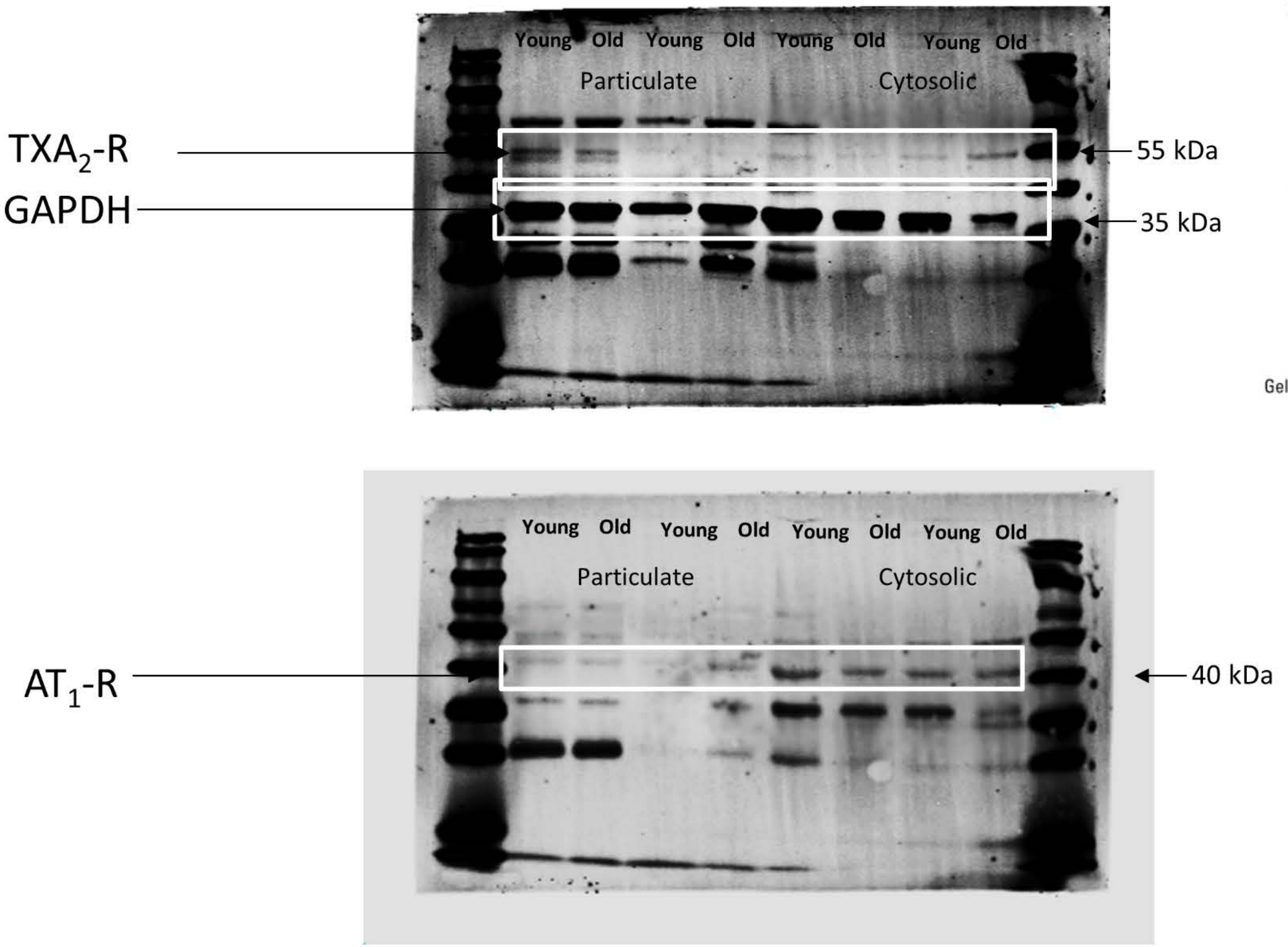

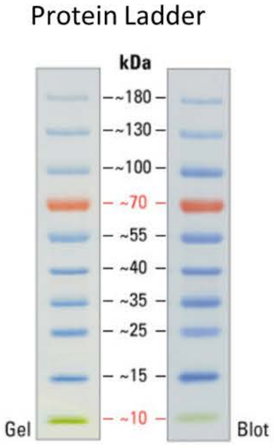

Figure S2. Full blot images of TXA_2-R (top panel) and AT_1-R (bottom panel) in young vs. old through western blot. Western blot analyses show significant decrease in particulate fraction of TXA_2-R and AT_1-R expression under aging. Values are compared with respect to GAPDH. Protein Ladder in the right panel represents the size standards for the full blot. The protein of interest is labelled with nearest ladder size to represent it's expected molecular weight.

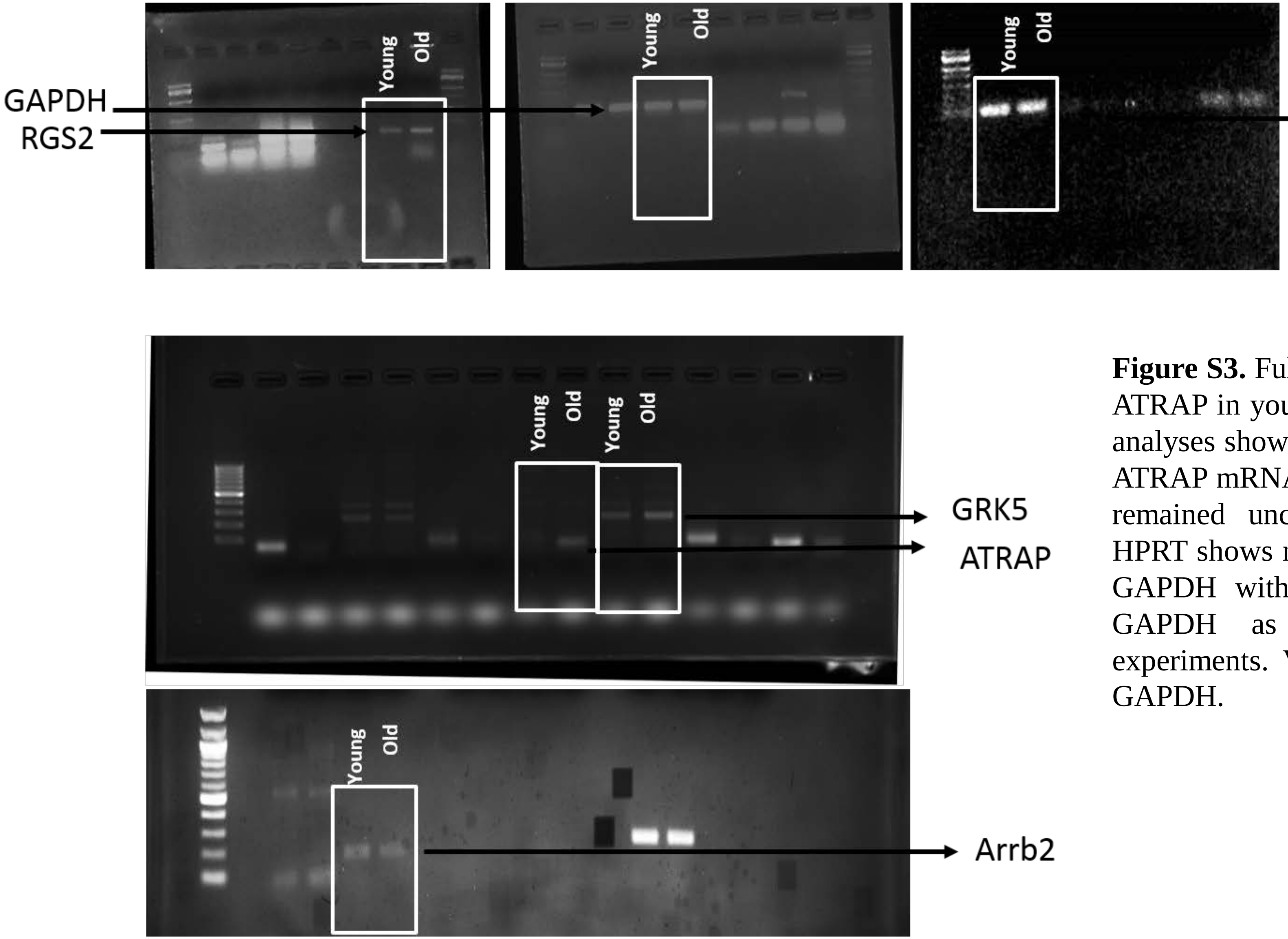

Figure S3. Full gel images of RGS2, Arrb2, GRK5, ATRAP in young vs. old through RT-PCR. RT-PCR analyses show significant increase in RGS2, GRK5, ATRAP mRNA expression under aging while Arrb2 remained unchanged under aging. RT-PCR for HPRT shows no significant change in expression of GAPDH with respect to HPRT, hence we used GAPDH as our control gene for further experiments. Values are compared with respect to GAPDH.

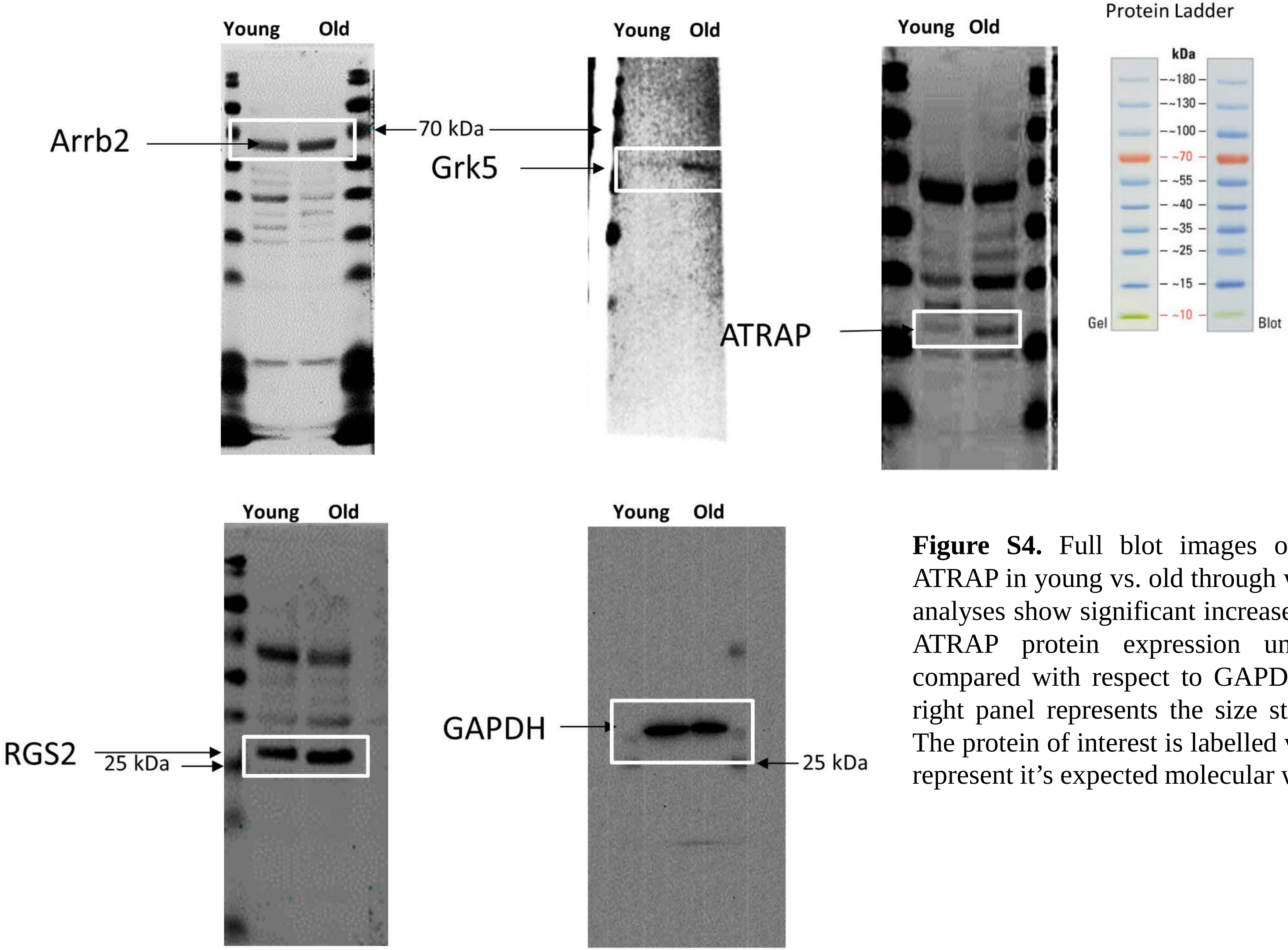

Figure S4. Full blot images of RGS2, Arrb2, GRK5, ATRAP in young vs. old through western blot. Western blot analyses show significant increase in RGS2, Arrb2, GRK5, ATRAP protein expression under aging. Values are compared with respect to GAPDH. Protein Ladder in the right panel represents the size standards for the full blot. The protein of interest is labelled with nearest ladder size to represent it's expected molecular weight.

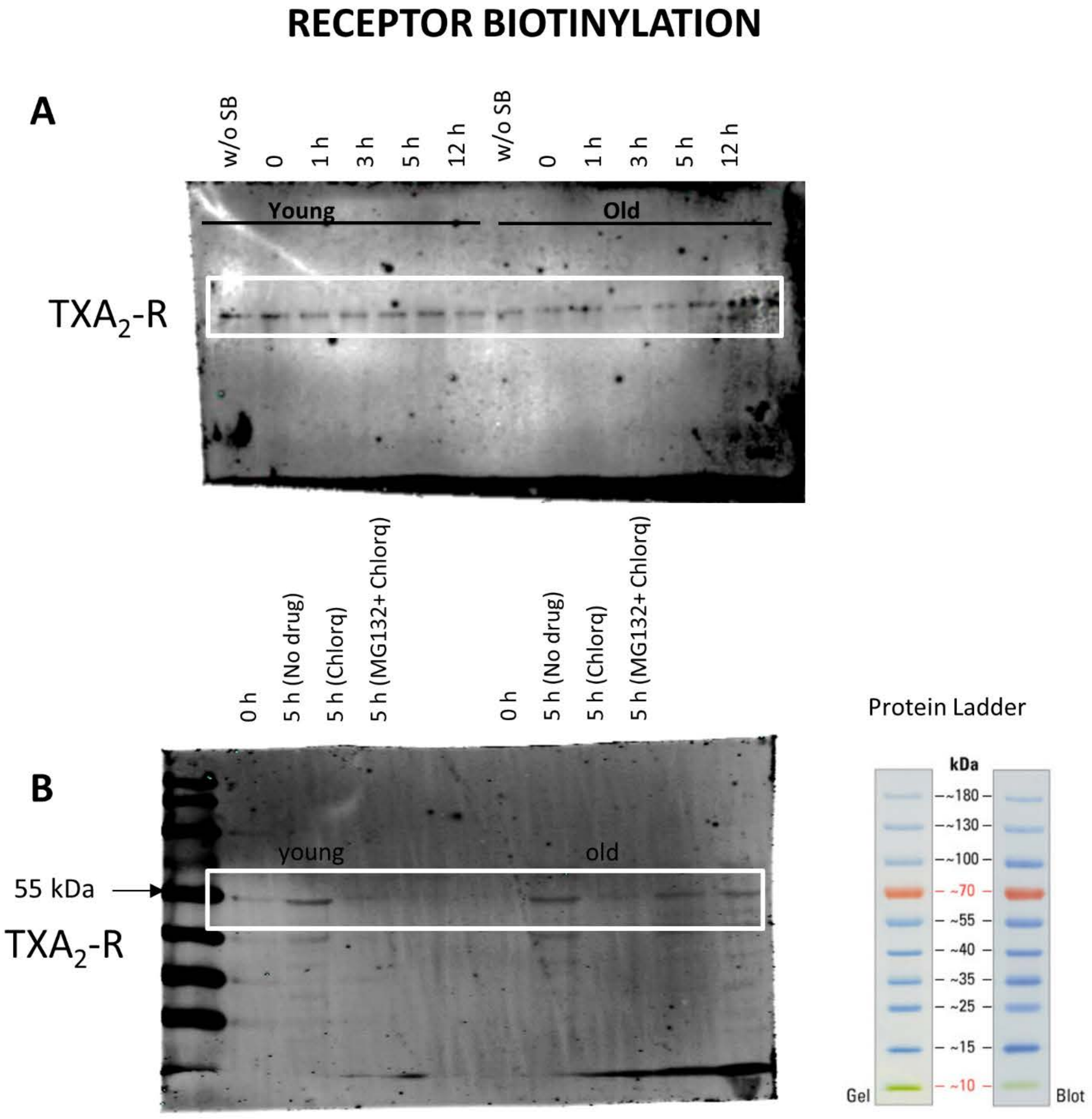

Figure S5. (A) Full blot images of TXA_2-R trafficking in young RIAS and old SMC by receptor biotinylation. Aging induces faster internalization at early phase of receptor trafficking (represented after 1h incubation) and slow recycling of cell-surface receptors. (B) Full blot images of TXA_2-R recycling rate in young vs old in presence of 10 μM MG132 (proteosomal inhibitor) and 10 μM chloroquine (lysomotrophic agent). Receptor density is calculated by normalizing the cell surface receptor density after various time points with respect to densitometry at 0 h. Protein Ladder in the right panel represents the size standards for the full blot. The protein of interest is labelled with nearest ladder size to represent it's expected molecular weight.
